# fastCCLasso: a fast and efficient algorithm for estimating correlation matrix from compositional data

**DOI:** 10.1093/bioinformatics/btae314

**Published:** 2024-05-10

**Authors:** Shen Zhang, Huaying Fang, Tao Hu

**Affiliations:** School of Mathematical Sciences, Capital Normal University, Beijing 100048, China; Beijing Advanced Innovation Center for Imaging Theory and Technology, Capital Normal University, Beijing 100048, China; Academy for Multidisciplinary Studies, Capital Normal University, Beijing 100048, China; School of Mathematical Sciences, Capital Normal University, Beijing 100048, China

## Abstract

**Motivation:**

The composition and structure of microbial communities on the body surface are closely related to human health. The interaction relationship among microbes can help us understand the formation of the microecological environment and the biological mechanism by which microorganisms influence host health. With the help of high-throughput sequencing technologies, microbial abundances in a natural environment can be directly measured without the isolation of microorganisms in culture. Sequencing experiments in microbiome studies can measure the relative abundance of microbes, which is called compositional data. Although there are already many methods for correlation analysis for compositional data, the computation time or accuracy still needs to be improved for current microbiome studies.

**Results:**

We develop a fast and efficient algorithm, called fastCCLasso, based on a penalized weighted least squares for inferring the correlation structure of microbes from compositional data in microbiome studies. We perform a large number of numerical experiments and the simulation results show that fastCCLasso outperforms its competitors in edge detection for inferring the correlation network. We also apply fastCCLasso for estimating microbial networks in microbiome studies and fastCCLasso provides a conservative network with comparable false discovery counts that are derived from shuffled data.

**Availability and implementation:**

FastCCLasso is open source and freely available from https://github.com/ShenZhang-Statistics/fastCCLasso under GNU LGPL v3.

## 1 Introduction

Each of us can be seen as a human superorganism that is a composite of microbes and human cells (Turnbaugh *et al.* 2007). The microbes living with us influence our health. Many studies have found that microbes are related to the health status of the host (McDonald *et al.* 2018). The diversity of microbial communities and the interaction relationship among microbes are fundamental to the microbial structure and the function on the host health (Estrela *et al.* 2015). Analysis of the microbial interaction network can help us to unravel the functional mechanisms of microbial communities on human health and disease.

High-throughput sequencing technologies such as 16S rRNA gene profiling (Kuczynski *et al.* 2012) provide an effective strategy for measuring microbial abundances in microbial communities. The microbial profiles are always represented by counts of operational taxonomic units (OTUs) in microbiome studies. Owing to the sequencing technical limitations such as the various sequencing depths, the count data from sequencing experiments only represents the relative abundance of microbes in the sample. The relative abundance data is called compositional data in statistics. It could cause spurious correlations if the traditional Pearson correlation coefficients are directly calculated from compositional data. In addition to the compositional effect, another challenge for the statistical inference in microbiome studies is the high dimensional feature that the number of OTUs is larger than the sample size.

Many computational and statistical methods have been proposed to infer the correlation network in microbiome studies. To overcome the compositional effect, some researchers treat the compositional data as the representation of unobserved absolute abundances, which are called basis variables. Friedman and Alm (2012) proposed an algorithm for the sparse correlations for compositional data (SparCC), which infers the correlations of basis variables from compositional data. One limitation of SparCC is its high computational complexity when the number of microbes is large. Watts *et al.* (2019) provided a parallelizable implementation of SparCC for solving the high-burden computational problem in the original version of SparCC. Another limitation of SparCC is the assumption that the average of correlations between one microbe and other microbes is approximately 0 and this assumption may be violated if there is a hub node and correlations between the hub node and other nodes are all positive. A more reasonable assumption is that the covariance matrix of basis variables is sparse. Under the sparse assumption, several computational methods have been proposed for inferring correlations from relative abundances. Fang *et al.* (2015) proposed an algorithm for correlation inference for compositional data through Lasso (CCLasso), which is based on the relationship between the covariance matrix of unobserved basis variables and that of the observed compositional matrix. The other two methods, the regularized estimation of the basis covariance based on compositional data (REBACCA) proposed by Ban *et al.* (2015) and the composition-adjusted thresholding method (COAT) proposed by Cao *et al.* (2019), are based on the log ratios between pairs of compositions. CCLasso, REBACCA, and COAT make use of the connection of linear transformations between the covariance matrix of basis variables and that of observed compositional data. The speed of COAT is faster than CCLasso and REBACCA since it uses an adjusted thresholding procedure instead of iterative optimization algorithms. COAT can be seen as a one-step approximate solution of a penalized least squares problem. Both CCLasso and REBACCA need to solve a Lasso problem in the high-dimensional setting when the number of microbes is large. The computational complexity is $O(p^{3})$ per iteration for CCLasso and $O(p^{4})$ for REBACCA where *p* is the number of microbes.

In this article, we develop a novel computational algorithm, called fastCCLasso, to infer the correlation network from compositional data in microbiome studies. FastCCLasso solves a penalized weighted least squares problem with the sparse assumption of the covariance matrix. Instead of the alternating direction method of multipliers in CCLasso, fastCCLasso introduces an auxiliary vector and provides a simple updating scheme in each iteration. FastCCLasso only involves the calculation of multiplications between matrices and vectors and avoids the eigenvalue decomposition and multiplications of large dense matrices in CCLasso. The computational complexity of fastCCLasso is $O(p^{2})$ per iteration. The performance of fastCCLasso is evaluated in both simulation studies and real microbiome datasets. The results show that fastCCLasso outperforms its competitors in edge detection and is faster than CCLasso. In real microbiome studies, fastCCLasso provides a conservative network and has comparable false discovery counts for the shuffled data.

## 2 Materials and methods

### 2.1 FastCCLasso

Suppose there are *p* components and $X={\left(X_{1},\ldots ,X_{p}\right)}^{T}$ is the observed p×1 compositional vector where $Z^{T}$ is the transpose of a matrix *Z*. Assume the corresponding basis vector is $Y={\left(Y_{1},\ldots ,Y_{p}\right)}^{T}$ that

$$X_{i}=Y_{i}/1_{p}^{T}Y,\;1\;\leq \;i\;\leq \;p,\;$$

where $1_{p}$ is the p×1 vector whose elements are all 1 s. In microbiome studies, *p* is the number of OTUs. Only the relative representation *X* can be measured in practice while the basis vector *Y* is not observed. Let lnZ be the logarithmic transformation by elements of a matrix *Z*, then the following linear transformation connects the unobserved absolute abundance *Y* and its compositional representation *X*,

$$\ln X=\ln Y-(\ln1_{p}^{T}Y)1_{p},\;$$

where the term $1_{p}^{T}Y$ is the unobserved total abundance of the basis variables. It is more convenient to deal with the correlation structure of basis variables in logarithmic scales than that of the original basis vector.

Let $F=I_{p}-1_{p}1_{p}^{T}/p$ where $I_{p}$ is the p×p identity matrix. Let Σ and $\tilde{\Sigma }$ be the covariance matrix of lnY and lnX, respectively. There is a linear relationship between Σ and $\tilde{\Sigma }$ as follows,

(1) $$\;\;F\Sigma F=F\tilde{\Sigma }F.$$

If $\tilde{\Sigma }$ is given, then more than one solution exists for Σ in Equation (1) since F is rank degenerate. For the model identification, Σ is assumed to be sparse that only a small proportion of off-diagonal elements of Σ is nonzero. Let *S* be the sample version of the covariance matrix $\tilde{\Sigma }$ for the observed compositional data, then a penalized weighted least squares estimator for the covariance Σ of the unobserved basic vector is to minimize the following objective function,

(2) $$\;\;\frac{1}{2}tr\left(AF(\Sigma -S)FBF(\Sigma -S)F\right)+\lambda ||\Sigma |{|}_{1,\text{off}},$$

where $tr(Z)=\sum_{i=1}^{p}Z_{ii}$ is the trace of a p×p matrix *Z*, $\left||\Sigma |{|}_{1,\text{off}}=\sum_{1\;\leq \;i\neq j\;\leq \;p}|\Sigma_{ij}\right|$ is the $\ell_{1}$ norm of the off-diagonal elements of Σ, λ is the tuning parameter that balances the loss function for model fitting and the penalty function for the sparse assumption on the covariance matrix, and the matrices A and B are two p×p diagonal matrices that put different weights on the elements of the residual matrix FΣF−FSF. The objective function in CCLasso is a special case in Equation (2) that $A=I_{p}$ and $B_{ii}=1/{\left(FSF\right)}_{ii},\;1\;\leq \;i\;\leq \;p$.

For developing a fast iterative procedure for estimating Σ, an auxiliary vector ω is introduced as follows,

(3) $$\;\;\omega =\frac{1}{p}\Sigma 1_{p}-\frac{1_{p}^{T}\Sigma 1_{p}}{2p^{2}}1_{p},\;$$

and we have $F\Sigma F=\Sigma -(\omega 1_{p}^{T}+1_{p}\omega^{T})$. Both Σ and ω can be treated as variables and an alternative objective function for Equation (2) is

(4) $$\;\;f(\Sigma ,\omega )=\frac{1}{2}tr\left(A\Delta B\Delta \right)+\lambda ||\Sigma |{|}_{1,\text{off}},$$

where

$$\Delta =\Sigma -(\omega 1_{p}^{T}+1_{p}\omega^{T})-FSF.\;$$

FastCCLasso solves the following optimization problem with a constraint $\Sigma =\Sigma^{T}$,

(5) $$\;\;(\hat{\Sigma },\hat{\omega })=\underset{\left(\Sigma ,\;\omega \right)}{\arg\min}f(\Sigma ,\omega ),\;$$

for deriving the estimator $\hat{\Sigma }$ for the covariance matrix Σ. The requirement for the two weight matrices A and B is that both of them should be diagonal matrices while fastCCLasso uses $A=I_{p}$ and $B_{ii}=1/{\left(FSF\right)}_{ii},\;1\;\leq \;i\;\leq \;p$ as the default. Instead of the positive definite constraint on Σ, fastCCLasso adopts the symmetrical constraint from the consideration of calculation time. Although the objective function in Equation (5) is derived by the expression of ω in Equation (3), the optimization in fastCCLasso relaxes the constraint in Equation (3) for getting a fast iteration algorithm.

The tuning parameter λ in Equation (4) can be chosen by a *K*-fold cross-validation procedure. First, all samples are equally split into *K* disjoint parts denoted as $P_{1},\ldots ,P_{K}$. Second, for each 1 ≤ i ≤ K, we calculate the sample version $S_{i}$ of $\tilde{\Sigma }$ from $P_{i}$, get the estimator $\left({\hat{\Sigma }}_{-i},{\hat{\omega }}_{-i}\right)$ of (Σ,ω) from $P_{1},\ldots ,P_{i-1},P_{i+1},\ldots ,P_{K}$, and compute the residual matrix ${\hat{\Delta }}_{i}={\hat{\Sigma }}_{-i}-({\hat{\omega }}_{-i}1_{p}^{T}+1_{p}{\hat{\omega }}_{-i}^{T})-FS_{i}F$. Thirdly, we calculate the cross validated error as $\text{CV}(\lambda )=\sum_{i=1}^{K}tr\left(A{\hat{\Delta }}_{i}B{\hat{\Delta }}_{i}\right)/(2K)$. Finally, $\lambda^{*}=\underset{\lambda }{\arg\min}\text{CV}(\lambda )$ is used as the final tuning parameter in fastCCLasso.

### 2.2 Optimization algorithm

We develop an efficient block coordinate descent algorithm for fastCCLasso as follows. Let $\left(\Sigma^{\left(k+1\right)},\omega^{\left(k+1\right)}\right)$ be the values for (Σ,ω) in the *k*th iteration. Then an iterative scheme for the optimization problem in Equation (5) is to solve the following two optimization problems,

$$\begin{array}{c} \omega^{\left(k+1\right)}=\underset{\omega }{\arg\min}f(\Sigma^{\left(k\right)},\omega ),\\ \Sigma^{\left(k+1\right)}=\underset{\Sigma }{\arg\min}f(\Sigma ,\omega^{\left(k+1\right)}). \end{array}$$

The objective function f(Σ,ω) is a quadratic function with respect to (w.r.t.) ω if Σ is fixed. Then the update for ω can be derived by setting the gradient of $f(\Sigma^{\left(k\right)},\omega )$ w.r.t. ω as 0 (Supplementary Text S1). The expression for updating ω is as follows,

$$\omega^{\left(k+1\right)}=C(I_{p}+D)C(B\Lambda^{\left(k\right)}A1_{p}+A\Lambda^{\left(k\right)}B1_{p}),$$

where $\Lambda^{\left(k\right)}=\Sigma^{\left(k\right)}-FSF$, C is a p×p diagonal matrix with

$$C_{ii}=\frac{1}{\sqrt{A_{ii}\sum_{j=1}^{p}B_{jj}+B_{ii}\sum_{j=1}^{p}A_{jj}}},\;1\;\leq \;i\;\leq \;p,$$

and

$$D=\frac{\left(b^{T}b)aa^{T}+(a^{T}a)bb^{T}-(1+a^{T}b)(ab^{T}+ba^{T}\right)}{{\left(1+a^{T}b\right)}^{2}-(a^{T}a)(b^{T}b)},$$

where *a* and *b* are two p×1 vectors that

$$a_{i}=C_{ii}A_{ii},\;b_{i}=C_{ii}B_{ii},\;1\;\leq \;i\;\leq \;p.\;$$

The update for Σ can be derived from the subgradient of $f(\Sigma ,\omega^{\left(k+1\right)})$ w.r.t. Σ (Supplementary Text S2) and the expression for updating Σ is as follows,

$$\begin{array}{c} \Sigma^{\left(k+1\right)}=\{\begin{array}{cc} G_{ij}^{\left(k+1\right)}, & \text{if}\;i=j,\\ G_{ij}^{\left(k+1\right)}-H_{ij}, & \text{if}\;i\neq j\;\text{and}\;G_{ij}^{\left(k+1\right)}>H_{ij},\\ G_{ij}^{\left(k+1\right)}+H_{ij}, & \text{if}\;i\neq j\;\text{and}\;G_{ij}^{\left(k+1\right)}<-H_{ij},\\ 0, & \text{otherwise}, \end{array} \end{array}$$

where $G^{\left(k+1\right)}=FSF+\omega^{\left(k+1\right)}1_{p}^{T}+1_{p}{\left(\omega^{\left(k+1\right)}\right)}^{T}$ and H is a p×p matrix with $H_{ii}=0,\;1\;\leq \;i\;\leq \;p$ and

$$H_{ij}=\frac{2\lambda }{A_{ii}B_{jj}+A_{jj}B_{ii}},\;1\;\leq \;i\neq j\;\leq \;p.\;$$

The above block coordinate descent algorithm always converges to a global optimum for the optimization problem in Equation (5) (Supplementary Text S3). The diagonal forms of A and B are crucial to derive simple forms for $\omega^{\left(k+1\right)}$ and $\Sigma^{\left(k+1\right)}$. Because of these simple expressions for updating, fastCCLasso avoids multiplications of dense matrices involved in CCLasso. The computational complexity of fastCCLasso is $O(p^{2})$ in each iteration for multiplications between matrices and vectors.

## 3 Results

### 3.1 Simulation studies

A series of simulation studies are conducted to compare the performance of fastCCLasso and its competitors including SparCC, CCLasso, and COAT. REBACCA is not used in simulations and real data analysis because of its high computational complexity when the dimension of variables is high. The tuning parameter, which balances the model fitting and the sparsity-inducing penalty, is selected by 3-fold cross-validation in fastCCLasso, CCLasso, and COAT. The default parameters are applied for SparCC.

The compositional data are simulated from the logistic multivariate normal distribution that lnY is generated from a multivariate normal distribution with a mean μ and a covariance matrix Σ. The mean vector μ is generated from a uniform distribution on ${\left[-0.5,0.5\right]}^{p}$ and the correlation matrix ρ, which is the standardization of Σ, is generated from one of six sparse network structures (Fang *et al.* 2017): random, neighbor, band, hub, block, and scale-free networks. The framework for simulating these six sparse networks is the same as Fang *et al.* (2017) except for settings of some parameters such as edge densities and connection strengths (Supplementary Text S4). Diagonal elements of Σ are generated from a uniform distribution on the interval *[*2, 5*]* and the covariance matrix Σ used in simulations is derived from the generated diagonals and the generated correlation matrix.

The primary interest in inferring a network structure is the edge detection whether an edge between two nodes is detected or not. We introduce three measures to evaluate the performance in edge detection: recall, precision, and F1 score. Recall is the fraction of correctly identified edges to the total number of true edges, which measures how well the true edges are correctly identified. Precision is the fraction of correctly identified edges to the total number of identified edges, which measures how much the identified edges are correct. Recall and precision are often negatively correlated that increasing recall always results in a decrease in precision, and vice versa. F1 score is the harmonic mean of precision and recall,

$$F1=\frac{2}{1/\text{precision}+1/\text{recall}},\;$$

which trades off the precision and the recall. We also consider two accuracy measures of estimations that

$$d_{1}(\hat{\rho },\rho )=\frac{||\hat{\rho }-\rho |{|}_{1,\text{off}}}{p(p-1)},\;\;\;d_{2}(\hat{\rho },\rho )=\sqrt{\frac{||\hat{\rho }-\rho |{|}_{F}^{2}}{p(p-1)}},$$

between the estimated correlation matrix $\hat{\rho }$ and the true correlation matrix ρ where $||Z|{|}_{F}=\sqrt{tr(ZZ^{T})}$ is the Frobenius norm of a matrix *Z*. Each simulation scenario is repeated 20 times and the average over these 20 replications for each method is used as the performance for comparisons.

We explore the performance of fastCCLasso with several settings for the weight matrices A and B in simulation studies. There is little impact on fastCCLasso for the choice of A and B while the default setting of fastCCLasso slightly outperforms others in most cases (Supplementary Fig. S1). We also check the influence on the positive definite of the covariance estimator that fastCCLasso uses a symmetric constraint instead of the positive definite constraint. FastCClasso always returns a positive definite estimator with the chosen tuning parameter in simulation studies (Supplementary Table S1).

We compare false discoveries for null networks with various combinations of the sample size and the dimension of variables that (n,p)=(300,50), (200,200), and (200,300). The diagonal elements of null networks are generated from the uniform distribution on *[*2, 5*]*. We filter correlation coefficients by an absolute threshold of 0.05 that if the absolute value of the inferred correlation coefficient between two variables is greater than 0.05, then these two variables are treated as connected. We find that there are almost no false discoveries for fastCCLasso, CCLasso, and COAT, but the percentage of false discoveries for SparCC is about 40% (Supplementary Table S2). We also explore the influence on the estimator of varying the filtering threshold of correlation coefficients. The performance of fastCCLasso has an elbow point at 0.05 for null networks (Supplementary Fig. S2) and fastCCLasso achieves its best performance at 0.05 in most cases for non-null networks (Supplementary Fig. S3). In the following simulation studies, the estimated correlation strengths for all methods are filtered by the threshold 0.05.

We explore the scenarios that the sample size is larger than the number of variables. Table 1 summarizes five measures of fastCCLasso, SparCC, CCLasso, and COAT for six different network structures with (n,p)=(300,50). Even though SparCC and CCLasso show higher recalls than fastCCLasso, the precisions and F1 scores of both SparCC and CCLasso are lower than that of fastCCLasso. This is due to the tendency to get a denser network estimation for SparCC and CCLasso, which results in wrongly identified edges. In contrast, fastCCLasso exhibits significantly higher precision values and F1 scores compared to SparCC, CCLasso, and COAT. Additionally, two accuracy measures $d_{1}$ and $d_{2}$ of fastCCLasso are also lower than that of other methods. In summary, fastCCLasso performs better than other methods in terms of edge detection.

**Table 1.** Performance comparisons of fastCCLasso, SparCC, CCLasso, and COAT for six network structures with (n,p)=(300,50).

Network	Method	Recall	Precision	F1	$d_{1}$	$d_{2}$
Random	fastCCLasso	0.841	0.799	0.815	0.033	0.061
	SparCC	0.980	0.323	0.485	0.072	0.080
	CCLasso	0.971	0.434	0.599	0.051	0.067
	COAT	0.859	0.693	0.767	0.035	0.061
Neighbor	fastCCLasso	0.984	0.651	0.782	0.058	0.078
	SparCC	0.995	0.359	0.527	0.110	0.107
	CCLasso	0.996	0.446	0.616	0.077	0.086
	COAT	0.973	0.340	0.504	0.136	0.127
Band	fastCCLasso	0.746	0.695	0.718	0.048	0.070
	SparCC	0.848	0.325	0.470	0.101	0.101
	CCLasso	0.854	0.439	0.579	0.067	0.079
	COAT	0.687	0.290	0.408	0.115	0.113
Hub	fastCCLasso	0.815	0.771	0.791	0.039	0.065
	SparCC	0.956	0.374	0.538	0.077	0.084
	CCLasso	0.944	0.465	0.623	0.059	0.073
	COAT	0.828	0.708	0.763	0.041	0.065
Block	fastCCLasso	0.762	0.704	0.730	0.051	0.071
	SparCC	0.830	0.314	0.456	0.118	0.111
	CCLasso	0.846	0.446	0.584	0.070	0.079
	COAT	0.767	0.281	0.411	0.144	0.129
Scale-free	fastCCLasso	0.517	0.595	0.550	0.055	0.085
	SparCC	0.869	0.324	0.472	0.087	0.094
	CCLasso	0.760	0.372	0.498	0.076	0.092
	COAT	0.461	0.359	0.403	0.074	0.097

Estimated correlation strengths for all four methods are truncated by the absolute threshold of 0.05. The results are the averages over 20 replications.

The situation that p ≥ n is also explored in simulation studies with (n,p)=(200,200) and (200,300) (Table 2). FastCCLasso performs better than other methods from precision values and F1 scores for random, neighbor, band, and block networks, while COAT performs best on the other two network structures and fastCCLasso is closely behind in second space. Specifically, the highest F1 scores for all methods are around 0.5 or even lower in some cases. This phenomenon may be due to the complexity of these network structures or weak edge strengths, which makes it difficult to detect edges. It is still a challenge to infer the correlation network from compositional data when the number of components is larger than the sample size in some network structures, especially for the hub and the scale-free networks.

**Table 2.** Performance comparisons of fastCCLasso, SparCC, CCLasso, and COAT for six network structures with (n,p)=(200,200) and (200,300).

Network	*p*	Method	Recall	Precision	F1	$d_{1}$	$d_{2}$
Random	200	fastCCLasso	0.878	0.773	0.817	0.004	0.027
		SparCC	1.000	0.020	0.040	0.088	0.096
		CCLasso	0.992	0.103	0.187	0.016	0.040
		COAT	0.906	0.689	0.782	0.004	0.026
	300	fastCCLasso	0.710	0.744	0.711	0.004	0.027
		SparCC	0.998	0.020	0.039	0.088	0.096
		CCLasso	0.957	0.119	0.211	0.014	0.037
		COAT	0.750	0.701	0.724	0.004	0.026
Neighbor	200	fastCCLasso	0.831	0.676	0.740	0.019	0.052
		SparCC	0.993	0.112	0.202	0.091	0.097
		CCLasso	0.970	0.219	0.357	0.044	0.068
		COAT	0.808	0.622	0.703	0.019	0.053
	300	fastCCLasso	0.778	0.666	0.703	0.013	0.044
		SparCC	0.994	0.077	0.143	0.089	0.096
		CCLasso	0.959	0.188	0.315	0.034	0.059
		COAT	0.752	0.657	0.701	0.013	0.045
Band	200	fastCCLasso	0.601	0.685	0.637	0.017	0.048
		SparCC	0.905	0.105	0.188	0.091	0.097
		CCLasso	0.803	0.210	0.333	0.039	0.064
		COAT	0.579	0.604	0.590	0.018	0.048
	300	fastCCLasso	0.563	0.696	0.620	0.012	0.041
		SparCC	0.909	0.072	0.134	0.090	0.097
		CCLasso	0.781	0.176	0.287	0.031	0.056
		COAT	0.555	0.639	0.594	0.012	0.041
Hub	200	fastCCLasso	0.873	0.736	0.797	0.004	0.028
		SparCC	0.999	0.023	0.044	0.088	0.096
		CCLasso	0.983	0.115	0.206	0.016	0.040
		COAT	0.877	0.696	0.775	0.004	0.027
	300	fastCCLasso	0.461	0.739	0.545	0.004	0.027
		SparCC	0.990	0.021	0.042	0.088	0.096
		CCLasso	0.725	0.111	0.189	0.012	0.036
		COAT	0.500	0.694	0.579	0.004	0.026
Block	200	fastCCLasso	0.540	0.651	0.588	0.060	0.085
		SparCC	0.807	0.338	0.477	0.113	0.111
		CCLasso	0.746	0.399	0.520	0.089	0.098
		COAT	0.518	0.545	0.530	0.068	0.092
	300	fastCCLasso	0.375	0.659	0.476	0.056	0.083
		SparCC	0.773	0.335	0.468	0.108	0.108
		CCLasso	0.658	0.409	0.504	0.081	0.093
		COAT	0.360	0.579	0.443	0.060	0.085
Scale-free	200	fastCCLasso	0.335	0.743	0.436	0.004	0.026
		SparCC	0.985	0.020	0.040	0.088	0.096
		CCLasso	0.695	0.124	0.210	0.010	0.033
		COAT	0.341	0.660	0.448	0.004	0.026
	300	fastCCLasso	0.426	0.764	0.492	0.003	0.023
		SparCC	0.994	0.014	0.027	0.088	0.096
		CCLasso	0.683	0.100	0.173	0.007	0.029
		COAT	0.480	0.704	0.569	0.003	0.022

Estimated correlation strengths for all four methods are truncated by the absolute threshold of 0.05. The results are the averages over 20 replications.

Table 3 shows the empirical computation time of fastCCLasso, SparCC, CCLasso, and COAT in the simulation studies. The computation is performed with a PC: Intel(R) Core(TM) i7-10700K @3.80 GHz CPU and 32 GB RAM. COAT is the fastest among the four methods since it uses an adjusted thresholding procedure for a one-step approximate solution instead of an iterating algorithm. The iteration framework of fastCCLasso is similar to that of CCLasso and the differences between these two methods are the objective function and the updating scheme. The empirical results show that fastCCLasso is at least 14 times faster than CCLasso.

**Table 3.** Running time (s) for six different network structures with (n,p)=(200,200) and (200,300). The results are the averages over 20 replications.

*p*	Method	Network structure					
		Random	Neighbor	Band	Hub	Block	Scale-free
200	fastCCLasso	4.4	5.8	5.4	5.6	8.3	4.1
	SparCC	1.2	1.2	1.2	1.6	1.2	1.2
	CCLasso	84.3	92.7	92.6	88.4	115.2	71.6
	COAT	1.1	1.1	1.1	1.3	1.1	1.1
300	fastCCLasso	8.4	11.1	10.1	9.6	14.4	7.9
	SparCC	3.0	3.2	3.1	3.9	3.1	2.9
	CCLasso	271.0	311.2	288.1	218.0	330.4	236.2
	COAT	2.3	2.5	2.4	2.7	2.4	2.3

We also compare fastCCLasso with other methods under non-Gaussian distributions via simulation studies. Multivariate *t*-distributions and linear transformations of uniform distributions for the logarithm scale of absolute abundances are used for testing the robustness of fastCCLasso under non-Gaussian assumptions (Supplementary Text S5). The conclusion in non-Gaussian cases is similar to that in the Gaussian case (Supplementary Tables S3 and S4).

CCLasso is a special case of fastCCLasso, but fastCCLasso outperforms CCLasso a lot in the above comparison. The reason is that the current implementation of CCLasso uses a bias-corrected procedure but this procedure brings about many false discoveries for node pairs without edges (Supplementary Table S5). We compare fastCClasso with the non-bias-corrected version of CCLasso. We find that the numeric performance of fastCCLasso is slightly worse than the non-bias-corrected version of CCLasso but fastCCLasso is faster than the non-bias-corrected version of CCLasso (Supplementary Table S6).

### 3.2 Analysis of microbiome data

FastCCLasso is applied to data analysis in two microbiome studies. The first dataset is a mouse skin microbiome data (Srinivas *et al.* 2013). Samples in this data belong to three groups: the immunized epidermolysis bullosa acquisita group (EBA), the immunized healthy group (Healthy), and the non-immunized control group (Control). This mouse skin microbiome data is filtered by removing OTUs represented in less than 60% samples and removing samples in which more than 60% OUTs are 0 s. 249 samples and 61 OTUs are used for the following analysis. These 249 samples include 58, 116, and 75 samples for EBA, Healthy and Control, respectively. The second dataset is the 16S rRNA sequencing samples from the American Gut Project (AGP) (McDonald *et al.* 2018). The microbiome data of body sites including skin, oral, and gut in AGP are considered in the following data analysis. The preprocessing of the count data in AGP is similar to the mouse skin microbiome data: the OTU count matrix is filtered by removing OTUs represented in less than 30% samples and removing samples in which more than 40% OUTs are 0 s. The sample sizes of skin, oral, and gut in AGP after data filtering are 331, 490, and 4829, respectively while the number of OTUs for skin, oral, and gut are 382, 256, and 436, respectively. All OTU counts are added by 0.5 and then normalized into compositions.

Since no prior information of true microbial interactions is known for the real microbiome data, we compare fastCCLasso with SparCC, CCLasso, and COAT from false discoveries by the shuffled OTU data, in which individual counts are permuted for each OTU. No microbial correlations are expected if network inference algorithms are applied to the shuffled OTU data. The shuffled procedure is repeated 20 times. Table 4 summarizes the percentage of false discoveries and the running time in the shuffled mouse skin microbiome data and the shuffled AGP data. SparCC returns many more false discoveries than other methods in the shuffled datasets. The false discoveries of fastCCLasso are acceptable compared to CCLasso and COAT. Moreover, fastCCLasso is much faster than CCLasso for the shuffled datasets under the same computational environment in simulation studies.

**Table 4.** Percentage (%) of false discoveries and running time (s) in the shuffled mouse skin microbiome data and the shuffled American Gut Project (AGP) data.

Method	Shuffled mouse skin data			Shuffled AGP data		
	EBA	Healthy	Control	Skin	Oral	Gut
False discoveries						
fastCCLasso	0.2	0.0	0.2	0.0	0.0	0.0
SparCC	70.4	59.5	66.7	36.3	26.8	0.1
CCLasso	2.2	0.0	0.8	0.0	0.3	0.0
COAT	0.1	0.0	0.1	0.0	0.0	0.0
Time (s)						
fastCCLasso	0.7	0.6	0.6	18.3	8.9	84.1
SparCC	0.1	0.1	0.1	8.4	3.3	13.1
CCLasso	2.9	2.1	2.7	410.8	115.5	435.1
COAT	0.1	0.1	0.1	4.8	2.2	13.1

The results are the averages over 20 replications.

**Figure 1.**
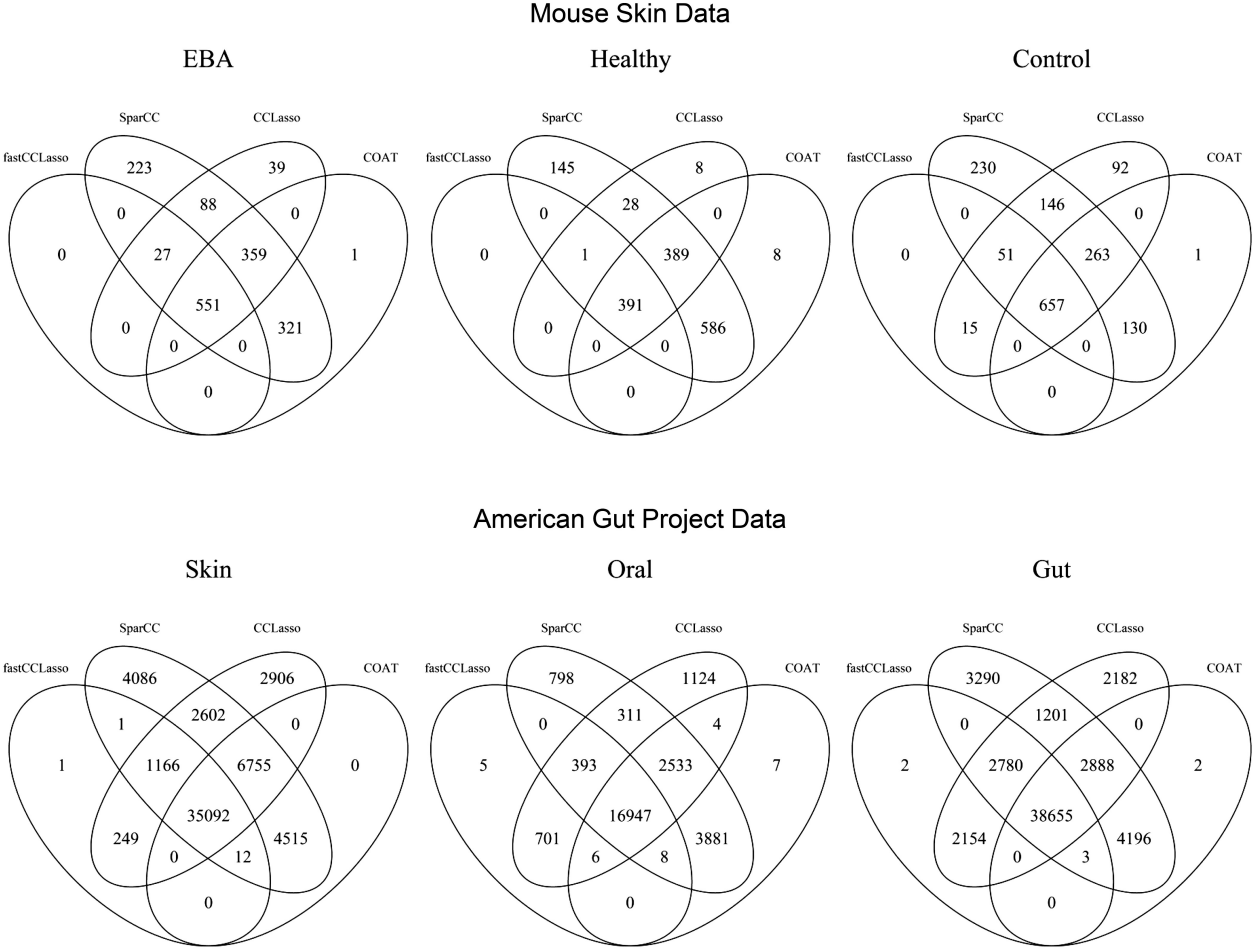
Overlaps of edges for networks inferred by fastCCLasso, SparCC, CCLasso, and COAT for the mouse skin microbiome data and the American Gut Project data.

The overlaps among networks for fastCCLasso, SparCC, CCLasso, and COAT for the original dataset are presented in Fig. 1. SparCC, CCLasso, and COAT tend to return a denser network, while fastCCLasso tends to return a sparser network and is relatively conservative to detect edges. The overlap of edges inferred by all four methods accounts for 25%−41% of all edges inferred by any one of four methods for the mouse skin data while this proportion is about 61%−67% for the AGP data. This difference may be attributed to the sample size of the AGP data being larger than that of the mouse skin data.

We also compare the robustness of inferred networks of all methods by the reproducibility and the Frobenius accuracy suggested by Fang *et al.* (2015) (Supplementary Text S6). Estimated correlation strengths for all methods are truncated by varying the threshold of edge strengths from 0 to 0.4. The corresponding reproducibility and the Frobenius accuracy of fastCCLasso with different edge thresholds are comparable to SparCC, CCLasso, and COAT (Supplementary Fig. S4).

## 4 Discussion

Inferring the interaction network of microbes is an important topic in microbiome studies since the microbial interaction pattern is closely related to the stabilization and evolution of a microbial ecosystem. We propose a fast and efficient computational method, called fastCCLasso, to infer the correlation structure of basis variables from the relative abundance observed in microbiome studies. The computational complexity of fastCCLasso is $O(p^{2})$ per iteration where *p* is the number of microbes while the computational complexities of its two competitors, CCLasso and REBECCA, are $O(p^{3})$ and $O(p^{4})$ per iteration, respectively. The simulation studies and data analysis of two real microbiome studies show that fastCCLasso performs well in edge detection for inferring the correlation network of basis variables from compositional data.

Even though fastCCLasso provides a fast computational scheme for inferring the correlation network from compositional data, there are still some challenges for network inference in microbiome studies. One challenge is the case when the number of microbes is much larger than the sample size. Simulation studies show that all methods considered in this work do not perform well in edge detection for hub networks and scale-free networks when the number of microbes is moderate. The second challenge is the zero-inflated feature for sequencing counts in microbiome data. Most computational methods including fastCCLasso are based on the logistical transformation of basis variables. The logistical transformation implies the observed 0 s are not “true” 0 s, but this is not always the case for microbiome data since some microbes don’t exist in some microbial environment. One possible extension is to consider the zero-inflated model for describing the “true” 0 s (Jiang *et al.* 2021; Zeng *et al.* 2023). The third challenge is the hierarchical structure of microbes that OTU data can have different levels of taxonomies. Zhou *et al.* (2021) incorporated the phylogenetic tree of OTUs into the statistical model for differential abundance analysis. Integrating the phylogenetic tree and the observed relative abundance of OTUs for inferring the correlation network is a promising research direction. These challenges point out possible improvements for network inference in microbiome studies.

## Supplementary Material

btae314_Supplementary_Data

## Data Availability

The data underlying this article are available in the article and in its online supplementary material.
