# Supplementary material for: fastCCLasso: a fast and efficient algorithm for estimating correlation matrix from compositional data
Source: Bioinformatics. 2024 May 10;40(5):btae314. doi: 10.1093/bioinformatics/btae314 (PMC11127107; doi:10.1093/bioinformatics/btae314)
Supplement: btae314_Supplementary_Data [file btae314_supplementary_data.pdf]

Supplementary Material for "fastCCLasso: Fast and efficient algorithm for estimating correlation matrix from compositional data"

Shen Zhang[1], Huaying Fang[2,3,*] and Tao Hu[1,*]

[1]School of Mathematical Sciences, Capital Normal University, Beijing 100048, China.

[2]Beijing Advanced Innovation Center for Imaging Theory and Technology, Capital Normal University, Beijing 100048, China.

[3]Academy for Multidisciplinary Studies, Capital Normal University, Beijing 100048, China.

[*]To whom correspondence should be addressed.

S1 Update ω with $\Sigma = \Sigma^{(k)}$

From $\Lambda^{(k)} = \Sigma^{(k)} - \mathbf{F}S\mathbf{F}$, the gradient of $f(\Sigma^{(k)}, \omega)$ with respect to (w.r.t.) ω is

$$\frac{\partial f(\Sigma^{(k)}, \omega)}{\partial \omega} = \mathrm{A}\left[\Lambda^{(k)} - \left(\omega \mathbf{1}_p^T + \mathbf{1}_p \omega^T\right)\right]\mathrm{B}\mathbf{1}_p + \mathrm{B}\left[\Lambda^{(k)} - \left(\omega \mathbf{1}_p^T + \mathbf{1}_p \omega^T\right)\right]\mathrm{A}\mathbf{1}_p.$$

Let $\partial f(\Sigma^{(k)}, \omega)/\partial \omega = 0$, then

$$\widetilde{\mathbf{H}}\omega = \mathrm{B}\Lambda^{(k)}\mathrm{A}\mathbf{1}_p + \mathrm{A}\Lambda^{(k)}\mathrm{B}\mathbf{1}_p,$$

where

$$\widetilde{\mathbf{H}} = (\mathbf{1}_p^T\mathrm{B}\mathbf{1}_p)\mathrm{A} + (\mathbf{1}_p^T\mathrm{A}\mathbf{1}_p)\mathrm{B} + \mathrm{A}\mathbf{1}_p(\mathrm{B}\mathbf{1}_p)^T + \mathrm{B}\mathbf{1}_p(\mathrm{A}\mathbf{1}_p)^T.$$

Since A and B are two $p \times p$ diagonal matrices whose diagonal elements are all positive, $\mathrm{A}\mathbf{1}_p$ and $\mathrm{B}\mathbf{1}_p$ are the diagonal elements of A and B, respectively. From the identity

$$\left(\mathbf{I}_p + z_1 z_2^T + z_2 z_1^T\right)^{-1} = \mathbf{I}_p + \frac{(z_1^T z_1) z_2 z_2^T + (z_2^T z_2) z_1 z_1^T - (1 + z_1^T z_2)(z_1 z_2^T + z_2 z_1^T)}{(1 + z_1^T z_2)^2 - (z_1^T z_1)(z_2^T z_2)},$$

where z_1 and z_2 are two $p \times 1$ vectors, the inverse of matrix $\widetilde{\mathbf{H}}$ has the following explicit expression,

$$\widetilde{\mathbf{H}}^{-1} = \mathrm{C}(\mathbf{I}_p + \mathrm{D})\mathrm{C},$$

where C is a $p \times p$ diagonal matrix with

$$\mathrm{C}_{ii} = \frac{1}{\sqrt{\mathrm{A}_{ii} \sum_{j=1}^{p} \mathrm{B}_{jj} + \mathrm{B}_{ii} \sum_{j=1}^{p} \mathrm{A}_{jj}}}, \; 1 \le i \le p,$$

and

$$\mathrm{D} = \frac{(b^T b) a a^T + (a^T a) b b^T - (1 + a^T b)(a b^T + b a^T)}{(1 + a^T b)^2 - (a^T a)(b^T b)},$$

where $a, \; b$ are $p \times 1$ vectors with $a_i = \mathrm{C}_{ii}\mathrm{A}_{ii}, \; b_i = \mathrm{C}_{ii}\mathrm{B}_{ii}$ for $1 \le i \le p$. Then the update for ω is

$$\omega^{k+1} = \widetilde{\mathbf{H}}^{-1}(\mathrm{B}\Lambda^{(k)}\mathrm{A}\mathbf{1}_p + \mathrm{A}\Lambda^{(k)}\mathrm{B}\mathbf{1}_p) = \mathrm{C}(\mathbf{I}_p + \mathrm{D})\mathrm{C}(\mathrm{B}\Lambda^{(k)}\mathrm{A}\mathbf{1}_p + \mathrm{A}\Lambda^{(k)}\mathrm{B}\mathbf{1}_p).$$

Computational complexity is mainly determined by the number of multiplication operations. In each iteration of fastCCLasso, the main computational complexity comes from the update for ω. The update for ω does not need to directly solve large linear equations. Since A, B and C are diagonal matrices and the calculation of D only involves the operation of vectors, the computational complexity in each iteration is determined by the multiplication between a diagonal matrix and a vector (computational

complexity $O(p)$) and the multiplication between a dense matrix and a vector (computational complexity $O(p^2)$). Therefore, the computational complexity in each iteration for fastCCLasso is $O(p^2)$.

S2 Update Σ with $\omega = \omega^{(k+1)}$

From

$$G^{(k+1)} = \mathbf{F}S\mathbf{F} + \omega^{(k+1)}\mathbf{1}_p^T + \mathbf{1}_p(\omega^{(k+1)})^T,$$

the objective function for Σ with $\omega = \omega^{(k+1)}$ is

$$\begin{aligned}
f(\Sigma, \omega^{(k+1)}) =& \frac{1}{2}\mathbf{tr}\left[\mathrm{A}(\Sigma - G^{(k+1)})\mathrm{B}(\Sigma - G^{(k+1)})\right] + \lambda\|\Sigma\|_{1,\text{off}} \\
=& \frac{1}{2}\sum_{i=1}^{p}\sum_{j=1}^{p}\left(\mathrm{A}_{ii}\mathrm{B}_{jj}\left(\Sigma_{ij} - G_{ij}^{(k+1)}\right)^2\right) + \lambda\sum_{1\le i\neq j\le p}|\Sigma_{ij}| \\
=& \sum_{1\le i<j\le p}\left[(\mathrm{A}_{ii}\mathrm{B}_{jj} + \mathrm{A}_{jj}\mathrm{B}_{ii})\left(\frac{\left(\Sigma_{ij} - G_{ij}^{(k+1)}\right)^2}{2} + \frac{2\lambda|\Sigma_{ij}|}{\mathrm{A}_{ii}\mathrm{B}_{jj} + \mathrm{A}_{jj}\mathrm{B}_{ii}}\right)\right] \\
&+ \frac{1}{2}\sum_{i=1}^{p}\left[\mathrm{A}_{ii}\mathrm{B}_{ii}\left(\Sigma_{ii} - G_{ii}^{(k+1)}\right)^2\right].
\end{aligned}$$

Then from $\Sigma^{(k+1)} = \arg\min_{\Sigma=\Sigma^T}\ f(\Sigma, \omega^{(k+1)})$ and the expression of H with $\mathrm{H}_{ii} = 0,\ 1 \le i \le p$ and

$$\mathrm{H}_{ij} = \frac{2\lambda}{\mathrm{A}_{ii}\mathrm{B}_{jj} + \mathrm{A}_{jj}\mathrm{B}_{ii}},\ 1 \le i \neq j \le p,$$

we have

$$\Sigma^{(k+1)} = \begin{cases} G_{ij}^{(k+1)}, & \text{if } i = j, \\ G_{ij}^{(k+1)} - \mathrm{H}_{ij}, & \text{if } i \neq j \text{ and } G_{ij}^{(k+1)} - \mathrm{H}_{ij} > 0, \\ G_{ij}^{(k+1)} + \mathrm{H}_{ij}, & \text{if } i \neq j \text{ and } G_{ij}^{(k+1)} + \mathrm{H}_{ij} < 0, \\ 0, & \text{otherwise.} \end{cases}$$

S3 Global convergence for fastCCLasso

The objective function $f(\Sigma, \omega)$ in Equation (4) is a convex function that is made up with two parts where the first part $f_0(\Sigma, \omega) = \frac{1}{2}\mathbf{tr}\,(\mathrm{A}\Delta\mathrm{B}\Delta)$ is a quadratic function w.r.t. (Σ, ω) and the second part $f_1(\Sigma) = \lambda\|\Sigma\|_{1,\mathrm{off}}$ is an ℓ_1 penalty function w.r.t the non-diagonal elements of Σ. The sublevels $\{(\Sigma, \omega)|f(\Sigma, \omega) \leq f(\Sigma^{(0)}, \omega^{(0)})\}$ is bounded and closed (compact) since $f(\Sigma, \omega) \to \infty$ as $\max(\max_{ij}|\Sigma_{ij}|, \max_i|\omega_i|) \to \infty$. There exists at least one minimum for $f(\Sigma, \omega)$. The iteration scheme of fastCCLasso is a block coordinate descent method. According to Theorem 5.1 in Tseng (2001), every cluster point generated by fastCCLasso is a coordinate-wise minimum point of $f(\Sigma, \omega)$. Let (Σ^*, ω^*) be one coordinate-wise minimum point of $f(\Sigma, \omega)$, then

$$\begin{aligned} f(\Sigma, \omega) - f(\Sigma^*, \omega^*) &= f_0(\Sigma, \omega) - f_0(\Sigma^*, \omega^*) + f_1(\Sigma) - f_1(\Sigma^*) \\ &\geq < f_{0,\Sigma}(\Sigma^*, \omega^*), \Sigma - \Sigma^* > + < f_{0,\omega}(\Sigma^*, \omega^*), \omega - \omega^* > + f_1(\Sigma) - f_1(\Sigma^*) \\ &= < f_{0,\Sigma}(\Sigma^*, \omega^*), \Sigma - \Sigma^* > + f_1(\Sigma) - f_1(\Sigma^*) \geq 0, \end{aligned} \tag{S.1}$$

where $f_{0,\Sigma}$ and $f_{0,\omega}$ is the gradient of f_0 w.r.t. Σ and ω, respectively and $< Z, W >= \mathbf{tr}(ZW^T)$ for two matrices Z and W. The inequality in the second line in Equation (S.1) is due to the convexity of $f_0(\Sigma, \omega)$. The equality in the third line in Equation (S.1) is from the coordinate-wise minimum in the direction of ω for $f(\Sigma, \omega)$ at the point (Σ^*, ω^*).

The inequality in the third line in Equation (S.1) is from the coordinate-wise minimum in the direction of Σ for $f(\Sigma, \omega)$ at the point (Σ^*, ω^*) that

$$0 \in f_{0,\Sigma}(\Sigma^*, \omega^*) + \partial f_1(\Sigma^*) \Rightarrow -f_{0,\Sigma}(\Sigma^*, \omega^*) \in \partial f_1(\Sigma^*)$$
$$\Rightarrow f_1(\Sigma) - f_1(\Sigma^*) \geq - < f_{0,\Sigma}(\Sigma^*, \omega^*), \Sigma - \Sigma^* >,$$

where $\partial f_1(\Sigma^*)$ is the subgradient of $f_1(\Sigma)$ at the point Σ^*. So every cluster point from fastCCLasso is one global minimum of $f(\Sigma, \omega)$.

S4 Generate six network structures

We generate six sparse network structures including random, neighbor, band, hub, block and scale-free networks. For $p = 50$, the generating procedure is given as follows.

1. **Random network**: For each pair of all p nodes, they are randomly connected with probability 0.2, and the strength of edges is set to ± 0.8 with equal probability.

2. **Neighbor network**: Randomly select p points as nodes from the region $[0, 1]^2$. Connect one node to its 10 nearest neighbors, and set the edge strength to 0.8.

3. **Band network**: Connect the ith node with the jth node if $i \neq j$ and $|i - j| \leq 6$. Set the edge strength to 0.4, 0.4, 0.2, 0.2, 0.1 and 0.1 as $|i - j| = 1, 2, 3, 4, 5$ and 6.

4. **Hub network**: Randomly select 3 nodes as hub nodes and denote the remaining nodes as non-hub nodes. Then connect each hub node to other nodes with the probability 0.4 and connect two non-hub nodes with the probability 0.2, and the strength of edges is uniformly generated from $[-0.8, -0.6] \cup [0.6, 0.8]$.

5. **Block network**: Divide p nodes into 5 blocks equally. Connect two nodes in the same block with the probability 0.9 and set the edge strength to 0.8. Connect two nodes in different blocks with the probability 0.1 and set the edge strength to 0.2.

6. **Scale-free network**: Apply the Barab$\acute{a}$si-Albert algorithm (Barabási and Albert, 1999) to build scale-free networks. Start with 5 full-connected nodes and then add 5 edges in each step. The edge strength is set to 0.9.

Both densities and strengths of networks are adjusted for the situation $p = 200$ and 300 to avoid weak connected networks. For the random network, the probability of connecting two nodes is set to 0.01; for the hub network, the probability of connecting each hub node to other nodes and the probability of connecting two non-hub nodes are set to 0.05 and 0.01, respectively; for the scale-free network, the number of edges added in each step is set to 1.

S5 Generate non-Gaussian distributions for latent variables

Let $\Sigma = LL^T$ be the Cholesky decomposition of the covariance matrix Σ. If the random vector W has the mean vector 0_p and the covariance matrix $\mathbf{I}_p$, then the linear transformation

$$\widetilde{W} = \mu + LW$$

can generate a random vector $\widetilde{W}$ with the mean vector μ and the covariance matrix Σ. The random vector can be used as the logarithm scale of the absolute abundance in simulation studies. We considered the standard Gaussian distributions for W in the main text and two non-Gaussian distributions in this supplementary material. The first

non-Gaussian distribution is the multivariate t-distribution, which is constructed with p independent and identically distributed random variables from t-distribution with degree 20 (with a scale for getting the unit variance). The second non-Gaussian distribution is derived from the uniform distribution on $[-\sqrt{3}, \sqrt{3}]$.

S6 Reproducibility and Frobenius accuracy in microbiome studies

The reproducibility and the Frobenius accuracy are used to measure the consistency of inferred networks from all data and from partial data. The correlation network structure estimated from all data is used as a reference. 90% of samples are randomly selected from the data to estimate the network structure. Estimated correlation strengths for all methods are truncated by different edge thresholds ranging from 0 to 0.4. The Frobenius accuracy is measured by the Frobenius norm distance between the estimate from all data and that from partial data. The reproducibility is measured by the fraction of shared edges between the estimator from all data and that from partial data.

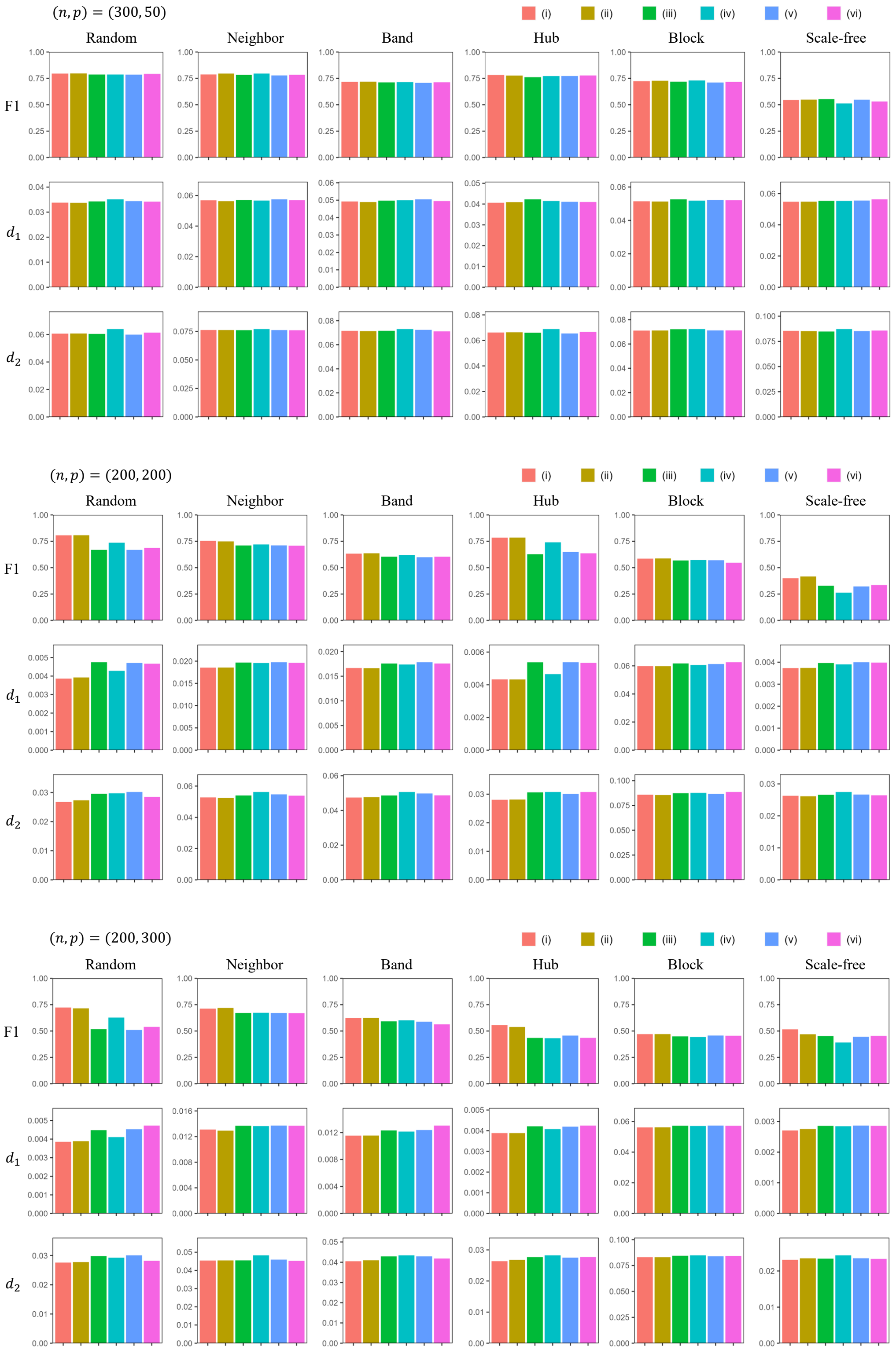

Fig. S1: Simulation results including F1 scores, d_1 and d_2 for various settings of A and B including (i) $\mathrm{A}_{ii} = 1$, $\mathrm{B}_{ii} = 1/(\mathbf{F}S\mathbf{F})_{ii}$, (ii) $\mathrm{A}_{ii} = 1$, $\mathrm{B}_{ii} = 1/S_{ii}$, (iii) $\mathrm{A}_{ii} = 1/(\mathbf{F}S\mathbf{F})_{ii}$, $\mathrm{B}_{ii} = 1/S_{ii}$, (iv) $\mathrm{A}_{ii} = \mathrm{B}_{ii} = 1$, (v) $\mathrm{A}_{ii} = \mathrm{B}_{ii} = 1/(\mathbf{F}S\mathbf{F})_{ii}$, (vi) $\mathrm{A}_{ii} = \mathrm{B}_{ii} = 1/S_{ii}$ in fastCCLasso. The results are the averages over 20 replicates.

Table S1: Proportion of how many output matrices are positive-definite for various tuning parameters λ in simulation studies. The proportion is calculated from 100 replicates. For the λ selected by cross-validation, all output matrices are positve-definite.

Network	n	p	λ									
			0.01	0.02	0.04	0.07	0.13	0.24	0.45	0.84	1.59	3.00
Random	300	50	1	1	1	1	1	1	1	1	1	1
	200	200	0	0.01	0.93	1	1	1	1	1	1	1
	200	300	0	0	0	1	1	1	1	1	1	1
Neighbor	300	50	1	1	1	1	1	1	1	1	1	1
	200	200	0.02	0.17	0.87	1	1	1	1	1	1	1
	200	300	0	0	0	1	1	1	1	1	1	1
Band	300	50	1	1	1	1	1	1	1	1	1	1
	200	200	0.06	0.66	1	1	1	1	1	1	1	1
	200	300	0	0	0	0.99	1	1	1	1	1	1
Hub	300	50	1	1	1	1	1	1	1	1	1	1
	200	200	0	0	0.84	1	1	1	1	1	1	1
	200	300	0	0	0.04	1	1	1	1	1	1	1
Block	300	50	1	1	1	1	1	1	1	1	1	1
	200	200	0	0.02	0.95	1	1	1	1	1	1	1
	200	300	0	0	0	0.99	1	1	1	1	1	1
Scale_free	300	50	1	1	1	1	1	1	1	1	1	1
	200	200	0.01	0.82	1	1	1	1	1	1	1	1
	200	300	0	0	0.29	1	1	1	1	1	1	1

Table S2: Percentage (%) of false discoveries that the strength of estimated edges is greater than 0.05 for the null network with various combinations of the variable dimension and the sample size. The results are the averages over 20 replications.

p	n	fastCCLasso	SparCC	CCLasso	COAT
50	300	0.0	39.7	0.0	0.1
200	200	0.0	48.0	0.0	0.0
300	200	0.0	48.1	0.0	0.0

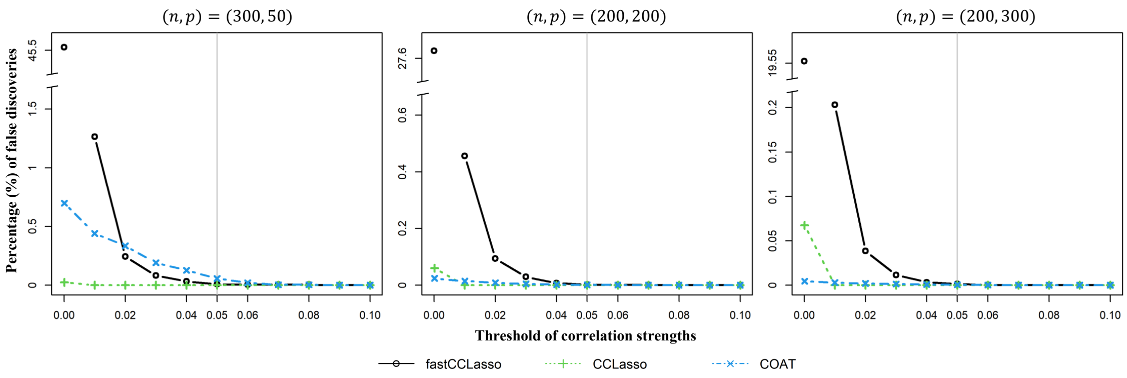

Fig. S2: False discoveries of fastCCLasso, CCLasso and COAT with varying filtering thresholds for null networks.

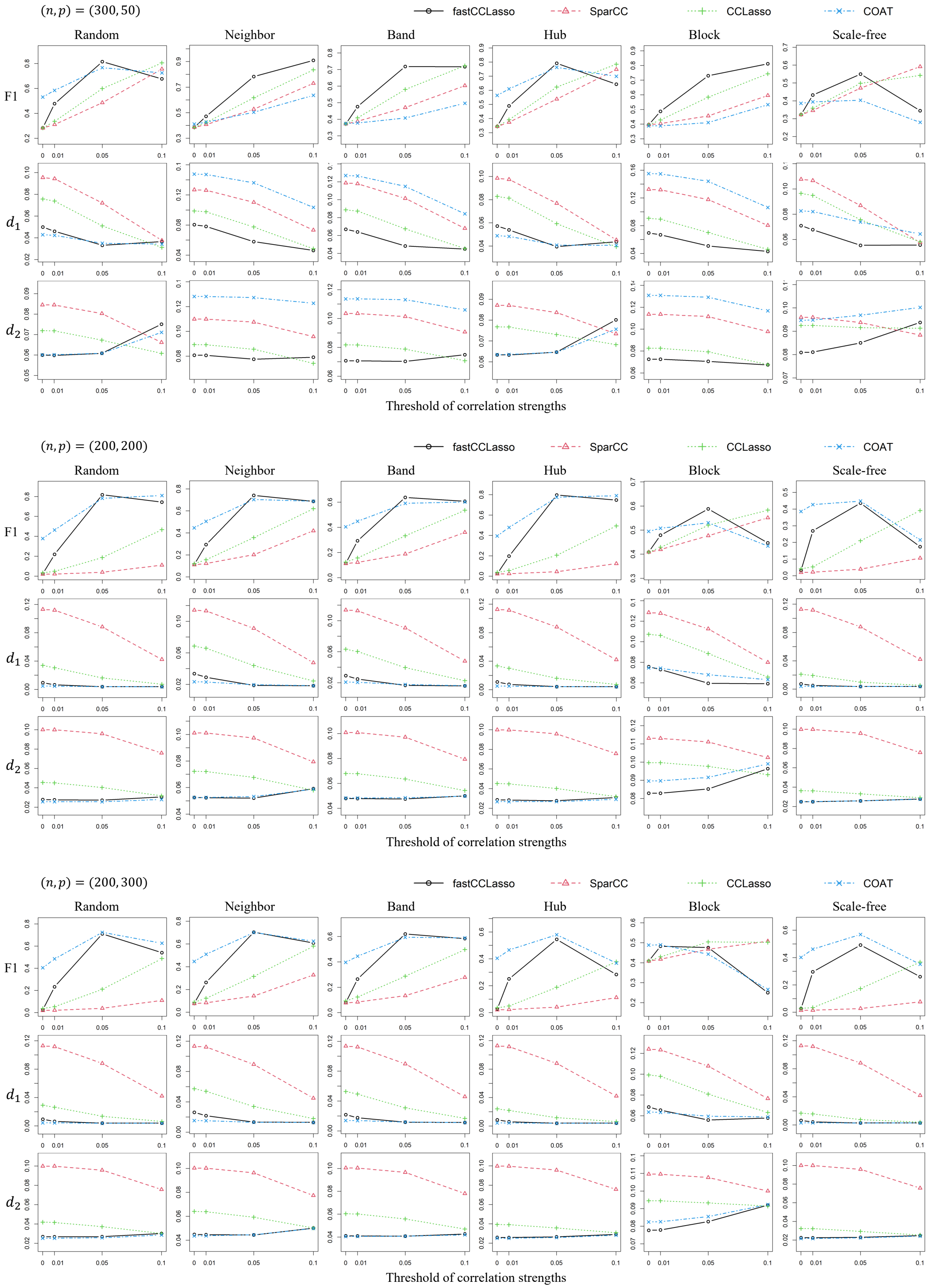

Fig. S3: Performance comparison (F1 scores, d_1 and d_2) of fastCCLasso and other methods with varying filter thresholds of correlation strengths for non-null networks.

Table S3: Performance comparisons of fastCCLasso, SparCC, CCLasso and COAT with the latent variables (logarithm scale) generated from the multivariate t-distribution. The results are the averages over 20 replications. The unit of computational time is the second.

n	p	Method	Random						Neighbor					
			Recall	Precision	F1	d_1	d_2	Time	Recall	Precision	F1	d_1	d_2	Time
300	50	fastCCLasso	0.824	0.741	0.779	0.035	0.063	0.8	0.976	0.632	0.767	0.063	0.083	1.5
		SparCC	0.971	0.304	0.463	0.079	0.086	0.1	0.994	0.357	0.526	0.113	0.110	0.1
		CCLasso	0.951	0.404	0.567	0.057	0.073	2.5	0.993	0.430	0.600	0.084	0.092	4.2
		COAT	0.826	0.662	0.734	0.038	0.064	0.1	0.959	0.347	0.509	0.135	0.129	0.1
200	200	fastCCLasso	0.840	0.737	0.775	0.004	0.028	4.3	0.785	0.661	0.702	0.020	0.055	5.2
		SparCC	1.000	0.019	0.038	0.095	0.101	1.2	0.993	0.108	0.194	0.098	0.103	1.1
		CCLasso	0.986	0.098	0.177	0.018	0.043	82.5	0.961	0.212	0.347	0.046	0.071	87.5
		COAT	0.878	0.677	0.764	0.004	0.027	1.1	0.780	0.615	0.687	0.020	0.055	1.0
200	300	fastCCLasso	0.644	0.747	0.683	0.004	0.028	9.2	0.672	0.681	0.642	0.014	0.048	9.7
		SparCC	0.997	0.019	0.037	0.096	0.102	3.0	0.991	0.073	0.137	0.097	0.102	2.8
		CCLasso	0.893	0.101	0.182	0.015	0.039	285.0	0.938	0.182	0.305	0.036	0.062	288.2
		COAT	0.700	0.691	0.694	0.004	0.027	2.4	0.702	0.632	0.664	0.014	0.047	2.3

n	p	Method	Band						Hub					
			Recall	Precision	F1	d_1	d_2	Time	Recall	Precision	F1	d_1	d_2	Time
300	50	fastCCLasso	0.727	0.687	0.704	0.051	0.074	1.1	0.781	0.750	0.764	0.042	0.068	0.9
		SparCC	0.854	0.321	0.466	0.106	0.105	0.1	0.953	0.363	0.526	0.082	0.089	0.1
		CCLasso	0.854	0.423	0.565	0.072	0.082	3.1	0.934	0.450	0.607	0.064	0.077	2.7
		COAT	0.687	0.300	0.417	0.113	0.113	0.1	0.804	0.689	0.741	0.043	0.068	0.1
200	200	fastCCLasso	0.570	0.654	0.607	0.018	0.050	4.8	0.820	0.696	0.748	0.005	0.029	4.5
		SparCC	0.896	0.100	0.179	0.098	0.103	1.1	0.998	0.022	0.042	0.096	0.102	1.2
		CCLasso	0.782	0.199	0.317	0.042	0.067	85.2	0.973	0.107	0.192	0.019	0.044	86.9
		COAT	0.552	0.587	0.568	0.019	0.051	1.0	0.831	0.677	0.745	0.004	0.028	1.1
200	300	fastCCLasso	0.522	0.727	0.605	0.012	0.044	9.2	0.376	0.732	0.467	0.004	0.028	9.0
		SparCC	0.906	0.069	0.128	0.097	0.103	2.8	0.987	0.020	0.039	0.096	0.102	3.0
		CCLasso	0.764	0.171	0.278	0.032	0.058	283.1	0.634	0.112	0.190	0.010	0.034	267.8
		COAT	0.531	0.640	0.580	0.012	0.042	2.2	0.442	0.670	0.530	0.004	0.027	2.4

n	p	Method	Block						Scale-free					
			Recall	Precision	F1	d_1	d_2	Time	Recall	Precision	F1	d_1	d_2	Time
300	50	fastCCLasso	0.759	0.677	0.714	0.054	0.074	1.2	0.464	0.607	0.515	0.057	0.087	0.9
		SparCC	0.836	0.312	0.455	0.122	0.115	0.1	0.858	0.307	0.452	0.092	0.098	0.1
		CCLasso	0.846	0.429	0.569	0.076	0.084	3.5	0.741	0.364	0.488	0.078	0.094	2.4
		COAT	0.764	0.291	0.421	0.142	0.130	0.1	0.415	0.367	0.388	0.072	0.097	0.1
200	200	fastCCLasso	0.504	0.641	0.560	0.062	0.089	7.0	0.196	0.794	0.297	0.004	0.027	3.7
		SparCC	0.808	0.333	0.471	0.118	0.116	1.1	0.978	0.019	0.038	0.095	0.102	1.0
		CCLasso	0.729	0.399	0.516	0.091	0.101	99.0	0.391	0.075	0.125	0.008	0.032	68.4
		COAT	0.482	0.545	0.510	0.069	0.095	1.0	0.280	0.656	0.389	0.004	0.026	1.0
200	300	fastCCLasso	0.347	0.648	0.449	0.057	0.085	13.2	0.365	0.739	0.447	0.003	0.024	8.0
		SparCC	0.774	0.327	0.460	0.115	0.113	2.9	0.993	0.013	0.026	0.096	0.102	2.8
		CCLasso	0.641	0.401	0.493	0.084	0.096	313.3	0.463	0.050	0.090	0.008	0.031	233.9
		COAT	0.332	0.577	0.419	0.060	0.087	2.3	0.427	0.658	0.515	0.003	0.023	2.3

Table S4: Performance comparisons of fastCCLasso, SparCC, CCLasso and COAT with the latent variables (logarithm scale) generated from the linear transformation of the multivariate uniform distribution. The results are the averages over 20 replications. The unit of computational time is the second.

n	p	Method	Random						Neighbor					
			Recall	Precision	F1	d_1	d_2	Time	Recall	Precision	F1	d_1	d_2	Time
300	50	fastCCLasso	0.845	0.769	0.802	0.034	0.061	1.1	0.988	0.647	0.781	0.056	0.075	2.0
		SparCC	0.979	0.317	0.479	0.074	0.082	0.1	0.996	0.356	0.525	0.110	0.108	0.1
		CCLasso	0.968	0.417	0.582	0.054	0.070	2.6	0.997	0.444	0.615	0.077	0.085	4.2
		COAT	0.871	0.657	0.748	0.036	0.061	0.1	0.979	0.335	0.499	0.138	0.128	0.1
200	200	fastCCLasso	0.898	0.727	0.789	0.004	0.027	4.6	0.829	0.674	0.741	0.019	0.052	5.7
		SparCC	0.999	0.020	0.040	0.088	0.096	1.3	0.994	0.112	0.202	0.091	0.097	1.2
		CCLasso	0.991	0.108	0.194	0.016	0.039	79.7	0.972	0.214	0.350	0.045	0.069	85.3
		COAT	0.919	0.696	0.792	0.004	0.025	1.1	0.805	0.628	0.705	0.019	0.053	1.1
200	300	fastCCLasso	0.710	0.744	0.708	0.004	0.027	9.1	0.771	0.676	0.713	0.013	0.045	10.4
		SparCC	1.000	0.020	0.039	0.089	0.096	3.1	0.993	0.077	0.142	0.090	0.097	3.0
		CCLasso	0.971	0.105	0.190	0.015	0.040	264.3	0.957	0.185	0.310	0.034	0.060	274.4
		COAT	0.770	0.690	0.727	0.004	0.025	2.4	0.751	0.653	0.698	0.013	0.045	2.4
n	p	Method	Band						Hub					
			Recall	Precision	F1	d_1	d_2	Time	Recall	Precision	F1	d_1	d_2	Time
300	50	fastCCLasso	0.752	0.677	0.711	0.049	0.070	1.4	0.834	0.751	0.789	0.040	0.064	1.3
		SparCC	0.845	0.325	0.469	0.102	0.102	0.1	0.961	0.375	0.540	0.077	0.084	0.1
		CCLasso	0.859	0.432	0.574	0.069	0.080	3.2	0.944	0.461	0.620	0.059	0.073	2.9
		COAT	0.696	0.294	0.413	0.115	0.113	0.1	0.837	0.698	0.761	0.041	0.064	0.1
200	200	fastCCLasso	0.587	0.697	0.632	0.017	0.048	5.1	0.838	0.739	0.765	0.004	0.028	4.6
		SparCC	0.906	0.105	0.188	0.091	0.097	1.2	1.000	0.023	0.044	0.088	0.096	1.3
		CCLasso	0.806	0.206	0.328	0.040	0.064	83.7	0.986	0.112	0.201	0.016	0.041	80.2
		COAT	0.573	0.610	0.591	0.018	0.048	1.1	0.875	0.699	0.776	0.004	0.026	1.1
200	300	fastCCLasso	0.557	0.717	0.624	0.012	0.041	9.5	0.441	0.773	0.552	0.004	0.027	9.0
		SparCC	0.910	0.072	0.133	0.090	0.097	3.1	0.990	0.021	0.042	0.088	0.096	3.1
		CCLasso	0.778	0.185	0.297	0.030	0.055	268.3	0.544	0.092	0.156	0.009	0.034	228.8
		COAT	0.549	0.651	0.595	0.012	0.041	2.3	0.511	0.689	0.585	0.004	0.026	2.4
n	p	Method	Block						Scale-free					
			Recall	Precision	F1	d_1	d_2	Time	Recall	Precision	F1	d_1	d_2	Time
300	50	fastCCLasso	0.770	0.676	0.719	0.051	0.070	1.7	0.538	0.593	0.562	0.055	0.084	1.2
		SparCC	0.832	0.316	0.458	0.118	0.112	0.1	0.871	0.317	0.465	0.089	0.095	0.1
		CCLasso	0.850	0.449	0.588	0.070	0.079	3.8	0.778	0.362	0.494	0.077	0.092	2.7
		COAT	0.765	0.282	0.412	0.143	0.129	0.1	0.482	0.358	0.410	0.075	0.097	0.1
200	200	fastCCLasso	0.556	0.638	0.590	0.060	0.085	8.4	0.303	0.779	0.412	0.004	0.026	4.1
		SparCC	0.808	0.339	0.477	0.112	0.111	1.2	0.986	0.020	0.040	0.088	0.096	1.2
		CCLasso	0.751	0.399	0.521	0.089	0.097	102.9	0.539	0.108	0.176	0.008	0.032	67.8
		COAT	0.529	0.540	0.533	0.068	0.091	1.1	0.338	0.656	0.442	0.004	0.026	1.1
200	300	fastCCLasso	0.396	0.655	0.491	0.055	0.082	14.4	0.465	0.730	0.506	0.003	0.023	8.2
		SparCC	0.777	0.336	0.469	0.108	0.107	3.1	0.995	0.014	0.027	0.088	0.096	3.0
		CCLasso	0.674	0.405	0.506	0.082	0.094	304.9	0.689	0.102	0.176	0.007	0.029	234.4
		COAT	0.377	0.576	0.455	0.059	0.085	2.4	0.489	0.693	0.572	0.003	0.022	2.3

Table S5: The percentage of non-zeros and the mean absolute error (MAE) for estimators of CCLasso without the bias-corrected procedure (CCLasso-0) and CCLasso with the bias-corrected procedure (CCLasso-1). The simulations are designed for the band network and the results are the averages over 20 replications.

		True correlation			
	CCLasso	0	0.1	0.2	0.4
Percentage of non-zeros	CCLasso-0	7.2 (2.3)	31.0 (10.2)	86.5 (6.6)	100 (0)
	CCLasso-1	32.6 (3.0)	59.4 (9.8)	96.5 (3.3)	100 (0)
MAE	CCLasso-0	0.005 (0.002)	0.078 (0.007)	0.283 (0.016)	0.079 (0.012)
	CCLasso-1	0.028 (0.003)	0.062 (0.007)	0.234 (0.014)	0.048 (0.007)

Table S6: Performance comparison of fastCCLasso, CCLasso without the bias-corrected procedure (CCLasso-0) and CCLasso with the bias-corrected procedure (CCLasso-1). The results are the averages over 20 replications. The unit of computational time is the second.

n	p	Method	Random						Neighbor					
			Recall	Precision	F1	d_1	d_2	Time	Recall	Precision	F1	d_1	d_2	Time
300	50	fastCCLasso	0.865	0.761	0.808	0.033	0.059	0.9	0.986	0.650	0.782	0.057	0.076	1.8
		CCLasso-0	0.840	0.809	0.823	0.032	0.060	1.5	0.986	0.722	0.833	0.048	0.070	2.5
		CCLasso-1	0.970	0.423	0.589	0.053	0.069	2.3	0.996	0.446	0.616	0.077	0.085	3.8
200	200	fastCCLasso	0.868	0.760	0.801	0.004	0.027	4.5	0.831	0.664	0.735	0.019	0.052	5.5
		CCLasso-0	0.843	0.844	0.841	0.004	0.027	50.7	0.784	0.765	0.773	0.018	0.053	56.3
		CCLasso-1	0.991	0.106	0.191	0.016	0.040	78.8	0.971	0.215	0.351	0.045	0.068	87.2
200	300	fastCCLasso	0.693	0.782	0.726	0.004	0.027	8.8	0.776	0.669	0.707	0.013	0.044	9.8
		CCLasso-0	0.674	0.829	0.742	0.004	0.027	170.8	0.699	0.797	0.743	0.012	0.046	174.9
		CCLasso-1	0.962	0.107	0.192	0.015	0.039	267.3	0.955	0.188	0.314	0.034	0.059	271.5

n	p	Method	Band						Hub					
			Recall	Precision	F1	d_1	d_2	Time	Recall	Precision	F1	d_1	d_2	Time
300	50	fastCCLasso	0.749	0.677	0.710	0.049	0.070	1.2	0.817	0.749	0.779	0.041	0.065	1.1
		CCLasso-0	0.742	0.730	0.735	0.045	0.068	1.9	0.788	0.795	0.791	0.039	0.066	1.6
		CCLasso-1	0.856	0.426	0.568	0.070	0.081	3.0	0.941	0.462	0.619	0.060	0.074	2.5
200	200	fastCCLasso	0.599	0.686	0.635	0.017	0.048	5.0	0.860	0.772	0.810	0.004	0.028	4.2
		CCLasso-0	0.563	0.781	0.653	0.016	0.047	54.4	0.818	0.851	0.833	0.004	0.028	51.4
		CCLasso-1	0.804	0.213	0.336	0.039	0.063	83.9	0.984	0.115	0.206	0.016	0.040	79.6
200	300	fastCCLasso	0.552	0.714	0.620	0.012	0.041	9.1	0.445	0.759	0.543	0.004	0.027	8.1
		CCLasso-0	0.529	0.792	0.633	0.011	0.041	174.6	0.283	0.635	0.389	0.004	0.028	145.8
		CCLasso-1	0.779	0.175	0.285	0.031	0.056	270.7	0.638	0.098	0.169	0.010	0.034	237.1

n	p	Method	Block						Scale-free					
			Recall	Precision	F1	d_1	d_2	Time	Recall	Precision	F1	d_1	d_2	Time
300	50	fastCCLasso	0.773	0.676	0.719	0.051	0.070	1.4	0.521	0.605	0.555	0.054	0.084	1.0
		CCLasso-0	0.774	0.737	0.755	0.045	0.065	2.3	0.514	0.640	0.568	0.053	0.084	1.5
		CCLasso-1	0.853	0.439	0.580	0.072	0.080	3.5	0.767	0.362	0.491	0.077	0.092	2.4
200	200	fastCCLasso	0.567	0.640	0.600	0.059	0.083	7.6	0.270	0.805	0.365	0.004	0.026	3.8
		CCLasso-0	0.533	0.714	0.610	0.055	0.082	65.8	0.172	0.507	0.252	0.004	0.027	40.6
		CCLasso-1	0.757	0.400	0.523	0.088	0.097	101.6	0.470	0.086	0.144	0.008	0.031	67.2
200	300	fastCCLasso	0.398	0.661	0.495	0.055	0.081	13.3	0.448	0.752	0.509	0.003	0.023	8.0
		CCLasso-0	0.356	0.721	0.475	0.054	0.082	195.2	0.313	0.626	0.411	0.003	0.024	141.8
		CCLasso-1	0.674	0.407	0.507	0.081	0.093	301.1	0.656	0.077	0.136	0.009	0.031	232.8

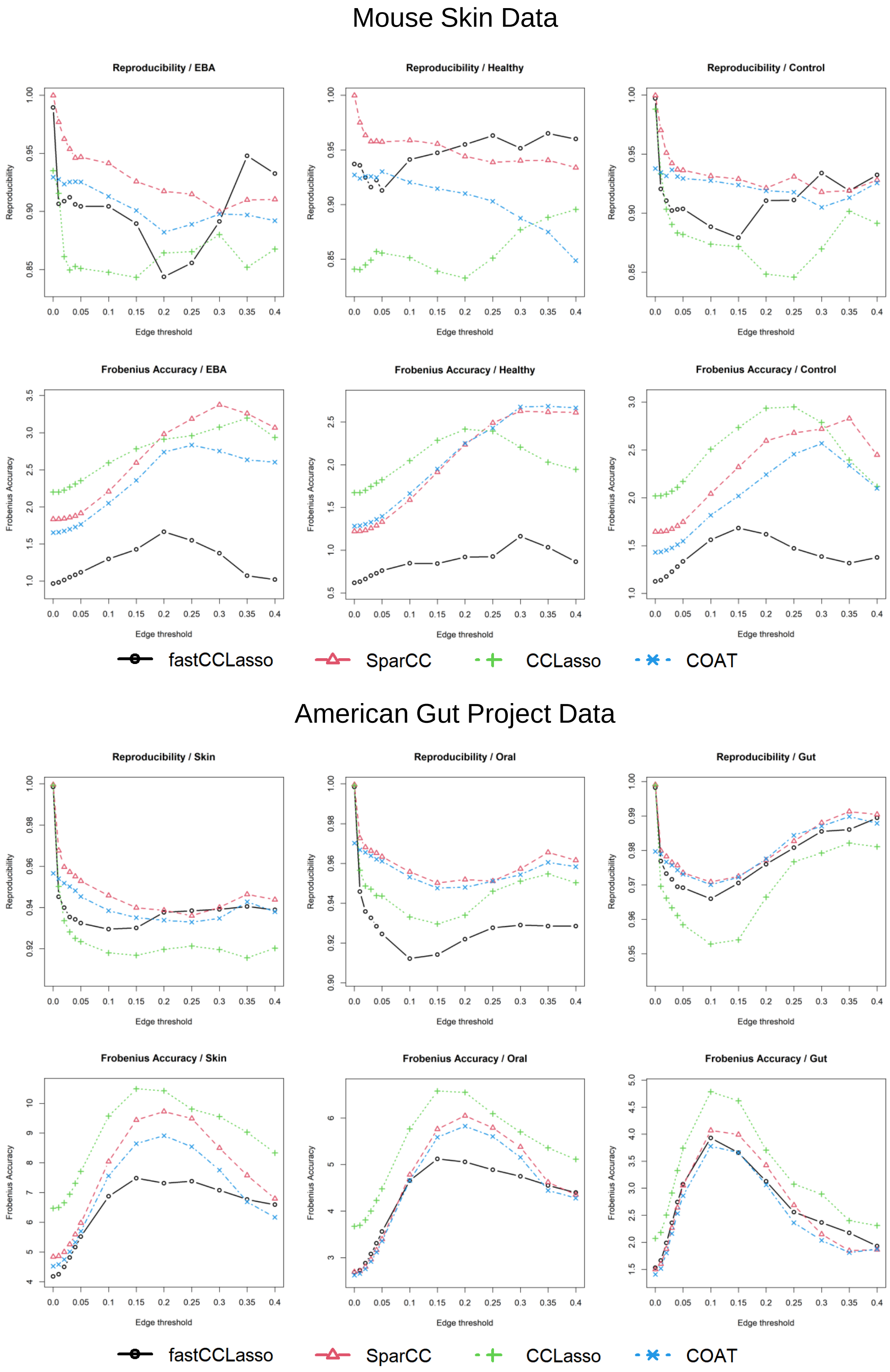

Fig. S4: Reproducibilities and Frobenius accuracies with varying thresholds of strengths of edges in the mouse skin microbiome data and the American Gut Project data.

References

Barabási, A.-L. and Albert, R. (1999). Emergence of scaling in random networks, *Science* **286**(5439): 509–512.

Tseng, P. (2001). Convergence of a block coordinate descent method for nondifferentiable minimization, *Journal of Optimization Theory and Applications* **109**(3): 475–494.
